# Bystanders’ reactions to animal abuse in relation to psychopathy, empathy with people and empathy with nature

**DOI:** 10.3389/fpsyg.2023.1124162

**Published:** 2023-05-12

**Authors:** Ana M. Martín, Andrea Vera, Rosario J. Marrero, Bernardo Hernández

**Affiliations:** ^1^Departamento de Psicología Cognitiva, Social y Organizacional, Universidad de La Laguna, San Cristóbal de La Laguna, Spain; ^2^Departamento de Psicología Clínica, Psicobiología y Metodología, Universidad de La Laguna, San Cristóbal de La Laguna, Spain

**Keywords:** animal abuse, dispositional empathy with nature, empathy, psychopathy, gender, environmental crime, pets, protected animal species

## Abstract

Social and academic interest in animal abuse has recently increased thanks to greater awareness of the importance of biodiversity in promoting sustainability. The redefinition of human-animal relationships, in the context of the fight against speciesism and the defense of veganism, has also contributed to this greater attention. Moreover, public awareness of animal rights has strengthened social reactions to violence against animals, though there are still some social sectors that are indifferent to these changes. Thus, better knowledge of the psychological mechanisms underlying reactions to animal abuse could contribute to better informal, social control of such abuse. The main aim of this study is to analyze the relationships between psychopathy, empathy with people, and empathy with nature arising from people’s reactions to protected and domestic animal abuse and illegal dumping. Also, as previous studies have shown differences between men and women, both in animal abuse and in personality traits, thus gender is taken into account when analyzing these relationships. A total of 409 people, resident in a highly environmentally protected territory, participated in this study. They were aged between 18 and 82 years old and 49.9% women. Participants were asked about assigned punishments, as well as the probability of intervening personally and/or calling the police, in relation to ten scenarios, based on press releases, describing one of three types of transgression of environmental laws: abuse of protected animals, abuse of domestic animals or illegal dumping. They also responded to Spanish adaptations of the Inventory of Callous Unemotional Traits, the Basic Empathy Scale, the Dispositional Empathy with Nature Scale, and the Social Desirability Scale. Each participant was randomly given ten scenarios corresponding to just one transgression type but all the personality scales. Results show that people’s reactions were greater for abuse of domestic than protected animals or for illegal dumping, irrespective of gender. Empathy with nature was related to the reaction against animal abuse more than empathy with people and psychopathy. Results are discussed highlighting the need for future research into similarities and differences between animal abuse and other types of environmental offences, which have many victims but no single being suffering.

## 1. Introduction

Social and academic interest in animal abuse has been increasing in recent years, mainly for two reasons. On the one hand, the importance of preserving biodiversity for the sustainability of the planet (Bruder et al., 2022). On the other hand, human-animal relationships are being redefined in the context of the fight against speciesism and the advocacy of veganism (Dhont et al., 2020). Public awareness of animal rights has also strengthened social reactions to violent behavior towards animals, though there are still sectors of society that remain unresponsive to these changes [see Bernuz and María (2022); https://www.mdsocialesa2030.gob.es/derechos-animales/index.htm]. Therefore, knowledge of the psychological mechanisms underlying the public’s reactions to animal abuse could contribute to a better informal, social control of such abuse. Especially as public awareness of a social problem often translates into a sense of responsibility and personal involvement that leads to interventions to help victims or prevent its occurrence (Gracia et al., 2018).

In academia, animal abuse has been defined as any intentional and unjustified behavior that involves causing suffering, harm, or pain to an animal (Ascione and Shapiro, 2009). Psychological research has primarily examined the relationship of animal abuse to other types of violence, such as intimate partner violence or domestic violence (Cleary et al., 2021). Studies have also investigated the ability to predict future violent behavior (Petersen and Farrington, 2007), as well as perpetrators’ personality traits such as psychopathy and empathy (Plant et al., 2019; Rock et al., 2021).

Studies into the relationship between animal abuse and personality traits have mostly focused on psychopathy (Rock et al., 2021), mainly on the callous-unemotional dimension (Dadds et al., 2006; Alleyne and Parfitt, 2019; Hartman et al., 2019). This dimension refers to the lack of guilt and/or remorse, emotional inexpressiveness, coldness in using others, insensitivity to others’ feelings, and lack of concern for one’s own task performance (Frick, 2009). High scores on callous-unemotional dimension have predicted animal abuse behaviors in both adult and child populations (Rock et al., 2021). However, whether this relationship is related to gender has not yet been studied, although significant differences have been found between men and women on all three traits of this dimension: callousness, uncaring and unemotional. Men usually score higher than women in all cases (Ciucci and Baroncelli, 2014). Given the relationship between animal abuse and psychopathic traits, it is to be expected that these traits may also be related to people’s reactions to animal abuse.

Another personality trait often associated with animal abuse is empathy with people (McPhedran, 2009). It has been proposed that empathy mediates the relationship between gender and attitudes towards animals (Graça et al., 2018). In general, individuals with high levels of empathy toward people are those who care more about the suffering of animals (Komorosky and O’Neal, 2015), especially if they are women, because women are more empathetic than men in relation to both humans and animals (Gómez-Leal et al., 2021). When the dimensions (cognitive and affective) of empathy are considered separately, it has been found that people who score higher in affective empathy, understood as an emotional response, are those who commit less animal abuse (Plant et al., 2019). In the study of Plant et al. (2019), women always scored higher than men in affective empathy, and those women who scored the highest were the ones that abused animals the least. In studies in which the cognitive and affective dimensions of empathy are compared, the results are inconsistent. Hartman et al. (2019) found that the cognitive dimension of empathy, understood as perspective taking, is linked to animal abuse, with those who score lower, abusing animals the most. However, in the study of Daly and Morton (2018), it is the lack of affective empathy that is associated with coldness and indifference towards animal and people in need of help. Empathy has been related to attitudes to animals and animal abuse but not yet to reactions to animal abuse, though it may be reasonable to expect such a relationship.

Most studies on empathy analyze the link between perpetrators’ empathy with people and animal abuse (e.g., McPhedran, 2009; Graça et al., 2018; Signal et al., 2018; Wied et al., 2021), but not the specific role of empathy with nature. This latter type of empathy, although it may be related to the first, refers to a different psychological construct (Paul, 2000; Gómez-Leal et al., 2021). Tam (2013) defines empathy with nature as “the dispositional tendency to understand and share the emotional experience of the natural world” (p. 93). The study of empathy with nature and the study of human-animal relationships have been closely related since their inception (Sevillano and Fiske, 2020). Therefore, it is to be expected that this personality trait may also be related to people’s reactions to animal abuse.

Research into animal abuse, in addition to the personality traits of perpetrators and observers, has also addressed the gender of both, and the type of animal victimized. Regarding perpetrators and observers’ gender, some studies show that men are more likely to engage in violent behavior towards animals than women (Plant et al., 2019; Zalaf and Egan, 2020; Kronhardt et al., 2021). Also women tend to assign harsher punishments to animal abusers than men (Signal et al., 2018) and have more pro-animalistic attitudes (Sims et al., 2007). Bailey et al. (2016) note that what predicts the punishment people assign to violent behavior towards animals is, besides the gender of the punisher, the type of animal victimized. Most of the studies on abuse have focused on domestic companion animals, mainly dogs and cats (e.g., Bailey et al., 2016). In general, people value the lives of domestic companion animals that are pets more highly than those they consider to be strays (Bailey et al., 2016). When human behaviors towards animals have been addressed in broader terms, other types of animals are considered. For example, there have been studies on farm animals focused on their welfare regarding red meat consumption and veganism and not animal abuse (e.g., Dhont et al., 2020). Protected animal species have also been the focus of attention regarding species trafficking crimes and the impact of climate change, technology and economic activity on biodiversity (e.g., Bruder et al., 2022). This evidence is along the same lines as research showing that human behavior towards animals varies according to the category of animal involved (Sevillano and Fiske, 2020). Therefore, it is expected that people’s reactions to animal abuse may be related to the type of animals involved.

At this point, it is worth noting that the Spanish Criminal Code (see https://www.boe.es/buscar/act.php?id=BOE-A-1995-25444) specifies differences among offenses against animals depending on the type of animal abused: protected species (Art. 334) and animals that are domestic or that temporarily or permanently live under human control (Art. 337). According to this law, animal abuse is an of the offense against environment described in Title XVI, and more specifically in Chapter IV, offenses relating to the protection of flora, fauna and domestic animals. Considering animal abuse as an environmental offense is not a trivial issue, not only from a legal but a psychological point of view, as it allows us to approach this behavior in relation to psychological variables already studied regarding compliance with environmental protection laws (Martín et al., 2014, 2017). Previous studies on environmental offenses have shown that people spontaneously distinguish between three categories of environmental transgressions when judging them: transgressions against the natural environment (including flora and fauna), construction activities and pollution/contamination (Martín et al., 2011). Therefore it is expected that people’s reactions against the abuse of both domestic and protected animals may be greater than against other types of environmental offenses in which the immediate victim is not a living being, such as illegal dumping.

Based on the evidence presented so far, the main aim of this study is to analyze the relationships between psychopathy, empathy with people and empathy with nature, and people’s reactions to the abuse of protected animals, to the abuse of domestic animals, and to illegal dumping. In addition, as previous studies have shown differences between men and women in both the reactions to animal abuse and in personality traits, gender will be considered when analyzing these relationships. Lastly, social desirability is included as a control variable given that, even in anonymous self-reports, people tend to give a positive image of themselves, especially when it comes to socially reproachable behavior like animal abuse (Glanville et al., 2021). More specifically, it is hypothesized that the reactions to the abuse of domestic animals will be greater than those to the abuse of protected animals and illegal dumping (H1), and these reactions will always be higher for women than for men (H2). It is also expected that the percentage of variance of the total reactions to transgressions explained by empathy with nature will be the greatest, compared to psychopathic traits and empathy toward people, in all three types of transgressions and both for men and for women (H3).

## 2. Materials and methods

### 2.1. Participants

A total of 409 people resident in the Canary Islands participated in this study. Age was between 18 and 82 years old (*M* = 31.04; *SD* = 13.26), and 49.9% were women. There were 46.5% with higher education, 41.5% had intermediate education and 11.9% were in compulsory education. In addition, 56.2% lived in an urban environment, 29.1% in a rural environment, and 14.7% in coastal areas of the same territory, which is highly protected by environmental law. All of them were resident in the study setting, since the catalogue of protected species varies among territories.

### 2.2. Instruments

*Scenarios describing transgressions of environmental protection laws*. Thirty scenarios describing environmental transgressions according to laws were prepared based on press releases. Ten of them were transgression against protected animals (e.g., “A hunter kills a kestrel by shooting it with his shotgun during a hunt”), ten transgressions against domestic animals (e.g., “A person leaves his dogs without food and water and lets them die on the roof”) and ten illegal dumping cases (e.g., “A company dumps pollutants down a drain into a ravine”). Different animals were included in the scenarios of both protected and domestic animal abuse (see Supplementary material). The thirty scenarios were selected from a pilot study in which forty-two scenarios were evaluated by 25 experts in relation to the degree in which these scenarios depicted anti-ecological behaviors, were imaginable, were likely to occur in their proximate surroundings and were described in an inclusive language.

Participants were randomly asked to rate the ten scenarios of one of the three types of transgression, indicating the severity of the punishment they would assign to the offender (“Culprit/s deserve/s punishment), how likely they would personally intervene to stop the transgression (“I would intervene personally”) and how likely they would call the police (“I would call the police”). Participants were asked to answer using an 11-point Likert-type scale from 0 = strongly disagree to 10 = strongly agree. The variables assigned punishment, personal intervention and call the police were calculated averaging participants’ answers to these questions in the ten scenarios of the transgression type they received at random.

The *Inventory of Callous Unemotional Traits* (ICU, Kimonis et al., 2008), in the Spanish adaptation by López-Romero et al. (2015) was used to measure the psychopathic traits of callousness, uncaring, and unemotional. This inventory consists of 24 items answered on an 11-point Likert-type scale from 0 = strongly disagree to 10 = strongly agree. These items are averaged to obtain a score on three subscales. The callousness subscale encompasses lack of empathy, guilt and remorse and consists of 11 items (e.g., “I do not care about hurting other people to get what I want”). The uncaring subscale measures the lack of concern for the feelings of others and the performance of one’s own tasks, and contains 8 items (e.g., “I always try to do my best”). The unemotional subscale refers to the lack of emotional expression and consists of 5 items (e.g., “I hide my feelings from others”). These three scales show adequate internal consistency in the Spanish adaptation by López-Romero et al. (2015): 0.76 for callousness, 0.82 for uncaring and 0.72 for unemotional.

The *Basic Empathy Scale* (Jolliffe and Farrington, 2006), in the Spanish adaptation by Villadangos et al. (2016) consists of 20 items answered on an 11-point Likert-type scale from 0 = strongly disagree to 10 = strongly Agree. These items are averaged to obtain a score on two subscales. The affective empathy subscale consists of 11 items (e.g., “Seeing a very angry person affects my feelings”) and refers to the ability to experience an emotional response similar to that of the other person. The cognitive empathy subscale consists of 9 items (e.g., “I understand my friends’ joy when something goes well for them”) and encompasses the rational ability to understand what other people feel. The internal consistency of the Spanish validation by Villadangos et al. (2016) was 0.92 for the affective empathy subscale and 0.96 for the cognitive empathy subscale.

The *Dispositional Empathy with Nature Scale* (Tam, 2013) was used in its Spanish adaptation by Sevillano et al. (2017). This scale measures the tendency to understand and share the emotional experience of the natural world and consists of 10 items (e.g., “I can imagine how I would feel if I were the animal or plant suffering”) that are answered on an 11-point Likert-type scale from 0 = strongly disagree to 10 = strongly agree. In the study by Sevillano et al. (2017), the reliability of the scale was 0.93.

The *Marlowe–Crowne Social Desirability Scale* (Crowne and Marlowe, 1960), in the short Spanish version by Gutiérrez et al. (2016), measures the tendency of participants to respond in a socially appropriate way. It consists of 18 items (e.g., “I always try to practice what I preach”) with dichotomous true (1) or false (0) responses that are summed to obtain a single final score. Internal consistency in the Gutiérrez et al. (2016) study was 0.78.

*Sociodemographic characteristics*. Participants were also asked about their gender, age, academic level and place of residence (rural, urban and costal).

### 2.3. Procedure

The instrument booklet was administered online via the Qualtrics^XM^ platform, through three links that were distributed at random to students of psychology and social work degrees. Each link was associated with the same instruments but included only one of the three type of scenarios describing transgressions of environmental protection laws. Each participant received randomly only one of these links. Students were asked also to disseminate the link to people of different genders and ages in their close environment and through social networks, using a “snowball” procedure. They were rewarded for their participation with extra points in a subject. At the beginning of the instrument booklet, the voluntary and anonymous nature of participation was stated, ensuring the confidentiality of the answers, and requesting express consent for participation. The presentation of the scenarios and the personality scales were randomized to control for a carry-over effect.

### 2.4. Design and data analysis

Two designs were used in this study (Ato et al., 2013). The first was a quasi-experimental-transversal design, with gender and transgression type as grouping variables and the reactions to the transgression variables, personal intervention, assigned punishment and call the police, as dependent variables. The second was a predictive-transversal design aimed at exploring a functional relationship by predicting the criterion variable total reaction to the transgression, from personality traits, such as predictors. The data were analyzed with the IBM SPSS Statistics 23.0 statistical package. First, the internal consistency of the reaction to the transgressions subscales, personal intervention, assigned punishment and call the police, and descriptive analyses of the variables resulting from averaging the items were calculated. Second, a MANCOVA was conducted, in which the criterion variable was gender, the dependent variables the reaction variables personal intervention, assigned punishment and call the police, and the covariate social desirability. Third, the internal consistency of the personality subscales, and descriptive analyses of the variables resulting from averaging the items of each subscale were calculated. Fourth, a MANCOVA was conducted, in which the criterion variable was gender, the dependent variables the scores on the personality subscales, and the covariate social desirability. Fifth, the variable total reactions to the transgressions were calculated by averaging the scores on the subscales assigned punishment, personal intervention and call the police, for each transgression type: Abuse of protected animals, abuse of domestic animals and illegal dumping. Before averaging, internal consistency and descriptive statistics for the new variable total reaction to the transgression were calculated. Finally, six stepwise multiple linear regression analyses were carried out to test which variables explained a higher percentage of variance of the total reaction to the transgression for each gender and for each type of transgression: abuse of protected animals, abuse of domestic animals and illegal dumping.

## 3. Results

The results obtained in relation to the internal consistency of the reaction subscales, personal intervention, assigned punishment and call the police, and descriptive analyses of the variables resulting from averaging the items are shown in Table 1. The internal consistency of all these subscales for all three transgression types was adequate.

**Table 1:** Internal consistency and descriptive analysis for the reaction variables personal intervention, assigned punishment and call the police according to transgression type.

	*α*	Min–Max	*M*	*DT*
Personal intervention				
Abuse of domestic animals	0.89	0–10	7.37	2.55
Abuse of protected animals	0.93	0–10	6.17	2.55
Illegal dumping	0.94	0–10	5.28	2.77
Assigned punishment				
Abuse of domestic animals	0.89	1–10	8.81	1.52
Abuse of protected animals	0.87	2.3–10	8.12	1.72
Illegal dumping	0.91	2.3–10	8.59	1.62
Call the police				
Abuse of domestic animals	0.90	0.2–10	7.02	2.38
Abuse of protected animals	0.93	0.4–10	6.40	2.68
Illegal dumping	0.93	0–10	5.99	2.78

In order to check H1 and H2, a MANCOVA was conducted with the reaction variables, personal intervention, assigned punishment and call the police, as dependent variables. The grouping variables were gender and transgression type, and social desirability the covariate. Multivariate effects were not statistically significant for the interaction between gender and transgression type (Pillai’s trace = 0.004, *F* (6, 796) = 0.28; *p* = 0.944, *η*^2^ = 0.002), nor for gender (Pillai’s trace = 0.012, *F* (3, 397) = 1.61; *p* = 0.185, *η*^2^ = 0.012). Social desirability (Pillai’s trace = 0.04, *F* (3, 397) = 5.47; *p* = 0.001, *η*^2^ = 0.04) and, after controlling this effect, transgression type (Pillai’s trace = 0.013, *F* (6, 796) = 9.49; *p* = 0.000, *η*^2^ = 0.067) had statistically significant effects. Therefore, H1 was confirmed and H2 rejected. As shown in Table 1, in general terms, the three types of reaction to the transgression were the highest for abuse of domestic animals, followed by abuse of protected animals and then by illegal dumping, regardless of gender. Tests of inter-subject effects regarding social desirability were statistically significant both for personal intervention (*F* (1, 399) = 6.20; *p* = 0.013, *η*^2^ = 0.015) and call the police (*F* (1, 399) = 12.19; *p* = 0.001, *η*^2^ = 0.03), but not for assigned punishment (*F* (1, 399) = 0.13; *p* = 0.910, *η*^2^ = 0.000). After controlling this effect, tests of inter-subject effects regarding transgression type were statically significant for personal intervention (*F* (2, 399) = 20.02; *p* = 0.000, *η*^2^ = 0.091), assigned punishment (*F* (2, 399) = 6.22; *p* = 0.002, *η*^2^ = 0.030) and call the police (*F* (2, 399) = 4.49; *p* = 0.012, *η*^2^ = 0.022). Pair comparisons (Sidak) showed statistically significant differences between the three transgression types for personal intervention. For call the police, statistically significant differences were found only between abuse of domestic and protected animals, and for punishment assignment between abuse of domestic animals and illegal dumping.

Next, before analyzing the impact of personality variables on total reaction to transgressions, gender differences were checked. The internal consistency of the personality traits subscales was adequate to calculate the personality variables by averaging the items. Alpha de Cronbach and descriptive analyses for these variables are shown in Table 2.

**Table 2:** Internal consistency and descriptive analysis for the personality variables.

	*α*	Min–Max	*M*	*DT*
Callousness	0.72	0.1–8.2	2.46	1.45
Uncaring	0.77	0–6.9	2.08	1.26
Unemotional	0.81	0–10	4.23	2.07
Cognitive empathy	0.81	3.33–10	7–45	1.36
Affective empathy	0.85	0.64–9.73	6.50	1.64
Empathy with nature	0.95	0–10	6.34	2.22
Social desirability	0.64	0–0.89	0.45	0.16

Internal consistency of all subscales was adequate, except in the case of callousness. After removing item 21 from this subscale, a satisfactory value was also obtained. In general, participants showed low scores on the different psychopathic-traits scales and moderate scores on the empathy scales.

A MANCOVA was carried out with the scores on the personality traits subscales as dependent variables, the grouping variable gender, and the covariate social desirability. Multivariate effects were statistically significant for social desirability (Pillai’s trace = 0.13, *F* (6, 398) = 9.94; *p* = 0.000, *η*^2^ = 0.13), and after controlling this effect, for gender (Pillai’s trace = 0.24, *F* (6, 398) = 21.04; *p* = 0.000, *η*^2^ = 0.24). Tests of inter-subject effects regarding social desirability were statistically significant only for the variable unemotional (*F* (1, 403) = 35.85; *p* = 0.000, *η*^2^ = 0.082), but differences due to gender remained statistically significant after controlling for this effect. Men and women differed in relation to callousness, uncaring and unemotional traits, cognitive empathy, affective empathy, and empathy with nature (see Table 3). Men scored higher than women on the psychopathic traits, while women scored higher on the cognitive and affective empathy and on empathy with nature.

**Table 3:** Comparison between men and women on personality variables.

	Women		Men		Inter-subject effects		
	*M*	*DT*	*M*	*DT*	*F* (1, 403)	*p*	*η* ^2^
Callousness	1.93	1.06	2.99	1.60	63.21	0.000	0.14
Uncaring	1.75	1.09	2.41	1.32	36.39	0.000	0.08
Unemotional	3.71	2.10	4.75	1.88	27.31	0.000	0.06
Cognitive empathy	7.94	1.10	6.97	1.42	59.77	0.000	0.13
Affective empathy	7.11	1.28	5.82	1.65	76.46	0.000	0.16
Empathy with nature	6.78	2.15	5.94	2.24	14.57	0.000	0.04

Although no gender differences were found in the reaction variables, they were in the personality variables. Therefore, to analyze whether the percentage of variance of the total reaction to the transgression explained by personality variables were different for men and women, six multiple regression analyses were carried out, three of them for women and three for men. In all cases, the predictors were the personality traits and the criterion variable was the total reaction to transgression. This variable was calculated by averaging the scores on the scales of assigned punishment, personal intervention and call the police for each transgression type: abuse of protected animals, abuse of domestic animals and illegal dumping. Table 4 shows Cronbach’s *α* values and descriptive statistics for this new variable.

**Table 4:** Internal consistency and descriptive statistics for the variable total reaction to transgression according to transgression type.

	*α*	Min–Max	*M*	*DT*
Total reaction to protected animal abuse	0.80	1.7–10	6.90	1.99
Total reaction to domestic animal abuse	0.79	1–10	7.73	1.84
Total reaction to illegal dumping	0.73	0.8–10	6.62	1.97

The correlations between social desirability, the criterion variable and the predictors, for men and women, are shown in Table 5. The results of the stepwise multiple linear regression analyses are shown in Table 6.

**Table 5:** Correlations between total reaction to transgression, personality variables and social desirability.

	Total reaction	Cognitive empathy	Affective empathy	Callousness	Uncaring	Unemotional	Empathy with nature	Social desirability
Total reaction	–	0.04^*^	0.19^**^	−0.01	−0.22^**^	−0.12	0.35^**^	0.14
Cognitive empathy	0.32^*^	–	0.42^**^	−0.26**	−0.48^**^	−0.22^**^	0.22^**^	−0.02
Affective empathy	0.18^*^	0.33^**^	–	0.24**	−0.43^**^	−0.34^**^	0.38^**^	−0.08
Callousness	−0.12	−0.35**	−0.19**	–	0.42^**^	0.21^**^	−0.03	0.01
Uncaring	−0.37^**^	−0.53^**^	−0.37^**^	0.37^**^	–	0.34^**^	−0.21^**^	−0.25^**^
Unemotional	−0.12	−0.23**	0.05	0.32^**^	0.23^**^	–	−0.20^**^	0.01
Empathy with nature	0.45^**^	0.34^**^	0.22^**^	−0.04	−0.33^**^	−0.11	–	0.04
Social desirability	0.16^*^	−0.17	−0.03	−0.18^*^	−0.34^**^	−0.06	0.09	–

The correlations in the lower part of the diagonal correspond to women and in the upper part to men. ^*^p < 0.05, ^**^p < 0.01.

**Table 6:** Results of the six stepwise multiple linear regression analyses of personality variables on the variable total reaction to transgression, according to transgression type and gender.

	Abuse of protected animals						Abuse of domestic animals						Illegal dumping					
	Women			Men			Women			Men			Women			Men		
	*R* ^2^_ajust._	*β*	*F* (2, 65)	*R* ^2^_ajust._	*β*	*F* (1, 66)	*R* ^2^_ajust._	*β*	*F* (1, 65)	*R* ^2^_ajust._	*β*	*F* (1, 21)	*R* ^2^_ajust._	*β*	*F* (2, 66)	*R* ^2^_ajust._	*β*	*F* (1, 65)
	0.31		16.17^***^	0.09		7.16^**^	0.22		19.41^***^	0.25		22.99^***^	0.21		10.06^***^	0.06		5.14^*^
Uncaring	−0.22^*^			–			–			–			−0.35^**^			−0.27^*^		
Empathy with nature	0.47^***^			0.31^**^			0.48^***^			0.51^***^			0.24^*^			–		

^*^p < 0.05, ^**^p < 0.01, ^***^p < 0.001.

As can be seen in Table 5, in the women’s group, social desirability correlated with total reaction to transgression, callousness and uncaring, while in the men’s group it only correlated with uncaring. The personality variable that correlated most strongly with total reaction to transgression was empathy with nature in both the men’s and women’s groups. Empathy with nature also correlated with cognitive empathy and affective empathy in both genders. For men, empathy with nature correlated negatively with the variables uncaring and unemotional traits, while for women it correlated only with uncaring.

Regarding the results of the stepwise multiple linear regression analyses (see Table 6), empathy with nature was the variable that explained a higher percentage of variance in the total reaction to the abuse of protected and domestic animals, while for illegal dumping it was uncaring. There were, however, some gender differences that deserve to be highlighted. In the men’s group, empathy with nature did not explain variance of illegal dumping, just uncaring. In the female group, uncaring was negatively related to both the total reaction to illegal dumping and total reaction to protected animal abuse. The remaining variables, cognitive empathy, affective empathy, callousness and unemotional traits, did not explain variance in the total reaction to the transgression in a statistically significant way in any of the transgressions. Social desirability did not influence these results, as it did not enter in any of the equations.

## 4. Discussion

The main objective of this study was to analyze the relationship between personality variables and people’s reactions to the abuse of protected and domestic animals and to illegal dumping, considering participants’ gender. The results showed, first, that there are statistically significant differences in reaction variables for the three transgression types (H1), irrespective of gender (H2). As expected, reactions were greater for domestic animals than for protected animals and illegal dumping, which did not differ significantly from each other, in line with previous studies (Sevillano and Fiske, 2020). This is probably because people feel more empathy with those animals they consider belong to them and, therefore, assign more punishment to violent behaviors directed towards domestic animals than towards other types of animals (Bailey et al., 2016).

Contrary to what was anticipated (H2), there were no gender differences in the reaction to transgression variables. Given the results from studies on psychopathic traits and empathy, and those obtained on attitudes towards animals, it was expected that women would react more negatively to anti-environmental behavior than men. Prior research has found that women have greater environmental concerns (Dietz et al., 2002) and more positive attitudes towards animals (Sims et al., 2007; Zalaf and Egan, 2015, 2017, 2020; Signal et al., 2018; Plant et al., 2019). It should be noted, however, that men show comparatively more positive attitudes towards wild animals than women, who prefer domestic animals (Bjerke et al., 1998). Gender-related differences were found in personality traits in line with previous research (Dadds et al., 2009; Ciucci and Baroncelli, 2014; Sevillano et al., 2017; Gómez-Leal et al., 2021). On the one hand, it was observed that men scored higher than women on the three psychopathic traits, so they tend to show less remorse and guilt, less interest in the feelings of others and in the performance of their own tasks, as well as little expression of their emotions. On the other hand, women scored higher on affective empathy, cognitive empathy, and empathy with nature, suggesting that they are better able than men to recognize the feelings of others and to understand and share the emotional experience of the natural world. However, when it comes to reactions to transgression variables, statistically significant differences between the two genders did not appear. One explanation for this lack of differences may be that the salience of environmental protection in the territory where the study was carried out, along the lines of the focus theory of normative conduct (Cialdini et al., 1991), has prevailed over the gender differences found in other contexts. In the studies by Martín et al. (2008, 2013) with samples of the general population in the same territory, gender differences were also not found when assessing several types of illegal anti-environmental behavior, including transgressions against protected fauna and flora. But before reaching definitive conclusions, future research should explore this issue by manipulating the impact of the salience of environmental protection laws on social reactions to animal abuse.

Regarding the relationship of personality variables with the variable total reaction to the transgressions, it was confirmed that the percentage of the variance of the criterion variable explained by empathy with nature were the greatest, compared to psychopathic traits and empathy toward people, in all three types of transgressions and both for men and for women (H3). Results also showed that, for women, the psychopathic traits of callousness, uncaring and unemotional correlated negatively with the total reaction to the transgressions, whereas cognitive empathy and affective empathy correlated positively, in line with previous research (Dadds et al., 2006; McPhedran, 2009; Alleyne and Parfitt, 2019; Plant et al., 2019). Thus, women who are more responsive to illegal anti-ecological behaviors are those who are better able to understand what others feel, to show concern for their feelings and to experience guilt and remorse. These findings are consistent with previous research indicating that animal abuse is negatively related to high scores on callousness-unemotional traits and lack of empathy (Daly and Morton, 2018).

However, when analyzing the explanatory power of personality variables regarding total reaction to anti-environmental behavior, taking into account transgression type and gender, there are nuances that are worth highlighting. Empathy with nature was the variable that explained the most variance in the case of domestic animals, in both men and women. But when it comes to illegal dumping, it is uncaring that explained a higher percentage of variance in both genders. These results suggest that the ability to understand and connect with the environment, including animals, is more influential in the face of animal abuse than the ability to put oneself in the place of other people. It is possible to show empathy with humans without necessarily showing empathy with animals, or vice versa (Gómez-Leal et al., 2021). Therefore, it is clear that the constructs of empathy with nature and empathy with people are related, but different, in line with what authors such as Paul (2000) and Tam (2013) point out. This finding highlights the need for future research on animal abuse in particular, and on environmental offences in general, to take into account the role of empathy with nature rather than empathy with people. It would also be interesting to analyze whether other approaches to people’s relationship with nature contribute to a better explanation of animal abuse. In this sense, besides empathy with nature, other constructs such as environmental identity (Clayton, 2003), implicit connection with nature (Schultz, 2001), or connectedness to nature (Mayer and Frantz, 2004) have been related to several pro-environmental behaviors (Carrus et al., 2015; Carmona-Moya et al., 2017; Pasca et al., 2022), in different contexts, including educational settings (Pirchio et al., 2021) and in different countries (Galharret et al., 2022).

Differences in the characteristics of the transgressions of animal abuse and illegal dumping can partially explain the differences in the relationship between the variables that provide the highest percentage of variance in total reaction to one transgression type and the other. Ecological offences such as illegal dumping, forest fires or illegal construction are characterized by the fact that, although many people may be affected, they are not associated with specific victims whose suffering is evident in the short term (Martín et al., 2014). In animal abuse, on the other hand, the consequences of the behavior on the victim may be immediately noticeable (Collado and Sorrel, 2019). Future research should, therefore, delve deeper into the behavioral specificity of ecological offenses, analyzing similarities and differences between animal abuse and other types of illegal anti-ecological behaviors.

This study has some limitations that should be considered when drawing conclusions from the results obtained. Animals included in the categories of protected and domestic animals have not been compared. People categorize animals in a similar way to humans, attributing specific characteristics to the members of each category and accommodating their perceptions and behaviors towards them according to their categorical membership (Sevillano and Fiske, 2020). Regarding domestic animals, it is possible that participants’ responses to dogs and cats, for example, are not the same as those to budgies or other pets. Also, there is evidence that participants react more strongly to the abuse of a bird than a reptile, even though both are protected species (Allen et al., 2002; Sevillano and Fiske, 2020). Another limitation that future research should address is that this study did not measure whether respondents had previously witnessed, or engaged in, any violent behavior against animals. Finally, given that the territory in which the study was carried out is a highly protected environment and home to a variety of protected flora and fauna species, the results obtained should be compared with others from areas where environmental policies are not so salient.

Despite these limitations, this work advances our knowledge on animal abuse from a psychological perspective, laying the groundwork for future research. The ultimate goal of this area of research is to develop evidence-based interventions to reduce animal abuse through informal, social control (Gracia et al., 2018). This would contribute to the sustainability of the planet (Balderjahn et al., 2013) through the preservation of biodiversity and a redefinition of human-animal relationships that is more in line with the present time.

## Data availability statement

The raw data supporting the conclusions of this article will be made available by the authors, without undue reservation.

## Ethics statement

The study was reviewed and approved by Comité de Ética de la Investigación y Bienestar Animal of the Universidad de La Laguna (CEIBA2022-3220). The participants provided their written informed consent to participate in this study.

## Author contributions

All authors listed have made a substantial, direct, and intellectual contribution to the work and approved it for publication.

## Funding

Funding from the Spanish Ministry of Science, Innovation (PID2021-122526NB-I00) and from the Universidad de La Laguna is acknowledged. AV has a research contract co-founded by the Canary Islands Agency for Research, Innovation and Information Society of the Regional Ministry of Economy, Knowledge and Employment and by the European Social Fund (ESF) Integrated Operational Programme of the Canary Islands, Axis 3 Priority Theme 74 (85%).

## Conflict of interest

The authors declare that the research was conducted in the absence of any commercial or financial relationships that could be construed as a potential conflict of interest.

## Publisher’s note

All claims expressed in this article are solely those of the authors and do not necessarily represent those of their affiliated organizations, or those of the publisher, the editors and the reviewers. Any product that may be evaluated in this article, or claim that may be made by its manufacturer, is not guaranteed or endorsed by the publisher.
