# Supplementary material for: Bystanders’ reactions to animal abuse in relation to psychopathy, empathy with people and empathy with nature
Source: Front Psychol. 2023 May 12;14:1124162. doi: 10.3389/fpsyg.2023.1124162 (PMC10213545; doi:10.3389/fpsyg.2023.1124162)
Supplement: Supplementary file 1 [file Data_Sheet_1.PDF]

SUPPLEMENTARY MATERIAL

Scenarios of abuse of protected animals.

- Un cazador mata a un cernícalo disparándole con su escopeta durante una cacería.
- Una embarcación recreativa persigue agresivamente a una manada de cachalotes durante una excursión.
- Una pareja da comida envenenada a un pinzón azul que había anidado en su ventana.
- Varios cazadores matan a un cuervo canario que se encontraba posado en un árbol.
- Un barco pesquero captura una foca monje para quedarse las aletas como trofeo.
- Una pareja tira piedras a un búho chico porque hacía ruido, hiriéndole en un ala.
- Una banda vende una aguililla canaria poniendo un anuncio a través de las redes sociales.
- Una persona hiere a una pardela tirándole una lata de cerveza mientras planeaba sobre el mar.
- Un pesquero captura a una tortuga boba que se encontraba cerca de la costa.
- Un grupo de personas cazan varias pardelas cenicientas para realizar una cazuela.

Scenarios of abuse of domestic animals.

- Varios cazadores abandonan a sus perros en el monte al finalizar la temporada de caza.
- Una familia se va de vacaciones y abandona a su gato en una gasolinera de la carretera.
- Una persona deja sin agua ni comida a sus perros, dejándoles morir en la azotea.
- Una persona deja morir a su gato por no llevarlo al veterinario para que le curen una
- herida.
- Unos vecinos asfixian a un perro callejero que se encontraba cerca de su domicilio.
- Una persona criadora de perros les corta las cuerdas vocales para que no hagan ruido.
- Una persona da una patada y lanza varios metros a un conejo que se le acercó en el monte.
- Varios amigos se graban en vídeo desplumando a un pájaro para subirlo a las redes sociales.
- Una persona le corta el pico a su pájaro para que no pueda cantar porque le molesta el ruido.
- Unos jóvenes abandonan en la carretera a un hámster que tenían en casa.

Scenarios of illegal dumping

- Una persona abandona su coche viejo en un espacio declarado Paraje Protegido.
- Un ayuntamiento permite que las aguas fecales, mal depuradas, de una urbanización se viertan al mar.

- Algunas personas del vecindario se deshacen de electrodomésticos viejos en un solar del barrio.
- Una persona se deshace de sus mascarillas quirúrgicas usadas tirándolas a un barranco.
- Una empresa vierte sustancias contaminantes por un desagüe que da a un barranco.
- Algunas personas del vecindario tiran escombros en un lugar declarado Parque Natural.
- La persona encargada de un taller se deshace del aceite de coche usado en un terreno cercano.
- Un taller de repuestos desecha los neumáticos viejos tirándolos en una playa cercana.
- Un grupo de jóvenes deja unas bolsas con basura en una playa de una Reserva Natural.
- Una persona abandona su coche viejo en un barranco tras comprarse uno nuevo.
